# A case of unilateral incomplete duplicated collecting system with ureteropelvic junction obstruction and renal pyelic fusion in a horseshoe kidney: A rare urinary tract malformation

**DOI:** 10.1016/j.eucr.2026.103515

**Published:** 2026-06-17

**Authors:** Yoshinori Ikehata, Naotaka Nishiyama, Hiroshi Kitamura

**Affiliations:** University of Toyama, Department of Urology, 2630 Sugitani, Toyama, 930-0194, Japan

**Keywords:** Horseshoe kidney, Duplicated collecting system, Pyelic fusion, Uretero-pelvic junction obstruction

## Abstract

A 2-month-old girl with congenital hydronephrosis was presented. Magnetic resonance urography confirmed the presence of a horseshoe kidney and right-sided duplicated collecting system. Retrograde pyeloureterography revealed right ureteral stricture and fusion between the left and right lower poles of the renal pelvis. Because of recurrence of urinary tract infection, pyeloplasty and vesicoureteral neostomy were performed at 14 months of age. No significant postoperative complications were observed, and the hydronephrosis improved over the course of approximately 1 year.

## Abbreviations:

VUR: vesicoureteral reflux
UPJO: uretero-pelvic junction obstruction

## 1 Introduction

Horseshoe kidney is the most common morphological abnormality of kidney fusion. It occurs in one in 400 people; however, most cases are asymptomatic.^1^ Among patients with horseshoe kidneys, in more than half of those with clinical symptoms such as febrile urinary tract infections, vesicoureteral reflux (VUR) or uretero-pelvic junction obstruction (UPJO) may occur. UPJO has been reported to occur in 13-34% of horseshoe kidneys.^2^ ^3^ Duplicated collecting systems are generally observed in 1% of urinary tract occurrences; however, their association with horseshoe kidneys is rare. Additionally, renal pyelic fusions are extremely rare.

Herein, we report a case of a horseshoe kidney with a unilateral incompletely duplicated collecting system with UPJO and renal pyelic fusion.

## 2 Case presentation

A 2-month-old female infant was referred to our institution from the pediatric department with a diagnosis of horseshoe kidney, bilateral hydronephrosis, and right renal atrophy. Magnetic resonance urography revealed the presence of a horseshoe kidney and right-sided duplicated collecting system (Fig. 1). Voiding cystourethrography revealed no VUR on either side. Diuretic renogram indicated an obstructive pattern in both the upper and lower poles of the right kidney. Delineation of the left kidney was poor, and its boundary with the right kidney was unclear. By 7 months of age, the patient had experienced multiple episodes of febrile urinary tract infections. We attempted to insert a ureteral stent; however, it failed to reach the renal pelvis. The right orifice was single, suggesting an incomplete duplication. Imaging of the left ureter confirmed that the guidewire had passed into the right lower renal pelvis, suggesting the presence of a fistula between the left and right renal pelvises. The flow at the fusion site was slow (Fig. 2).

**Fig. 1:**
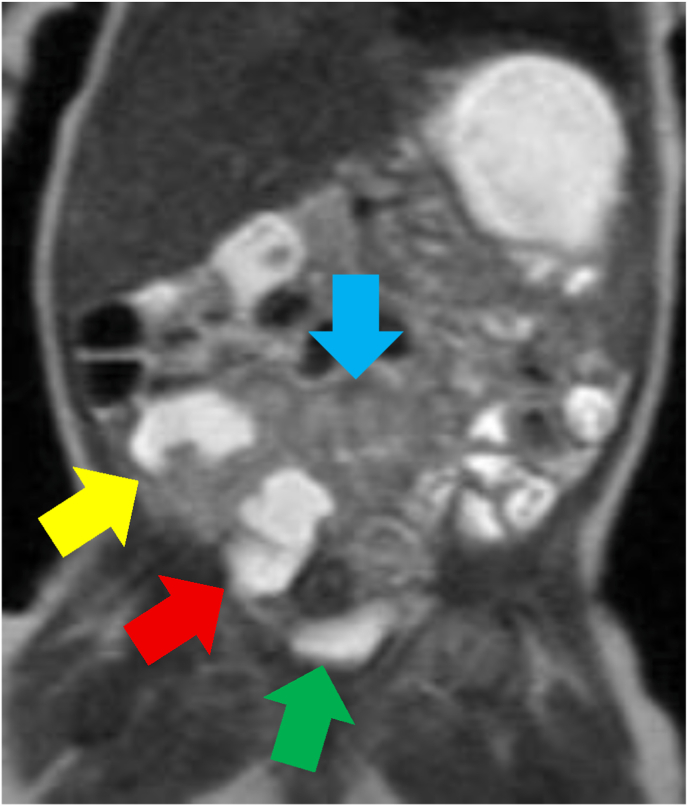
Magnetic resonance urography at the first visit. A horseshoe kidney with duplicated collecting system on the right side was suspected. Yellow arrow: upper pole pelvis of the right kidney. Red arrow: Lower pole of the pelvis of the right kidney. Green arrow: bladder. Blue arrow: left kidney.

**Fig. 2:**
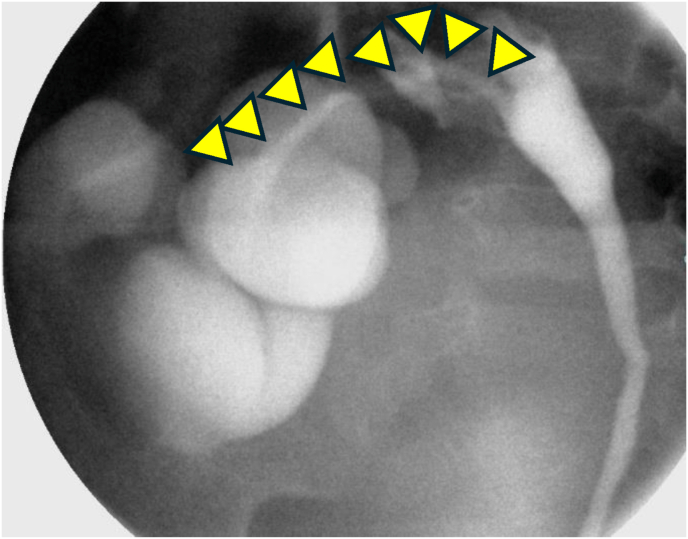
Retrograde pyeloureterography findings. It indicated communication between the left kidney and lower pole of the right kidney. Yellow arrow: pathway of contrast flow from the left renal calyx.

At 14 months of age, the patient underwent pyeloplasty and vesicoureteral anastomosis. Intraoperative findings are shown in Fig. 3. The ureter originating from the upper kidney passed in front of the renal pelvis of the lower kidney and joined the renal pelvis, and the ureter at the junction was stenotic. Additionally, the ureter originating from the lower kidney was stenotic at the uretero-pelvic junction and very narrow throughout its length. The ureter of the lower kidney was disconnected from the bladder and anastomosed end-to-side with the ureter of the upper kidney. The unified ureter was re-anastomosed with the bladder, and a ureteral stent was placed. A nephrostomy catheter was placed in the renal pelvis of the lower kidney.

**Fig. 3:**
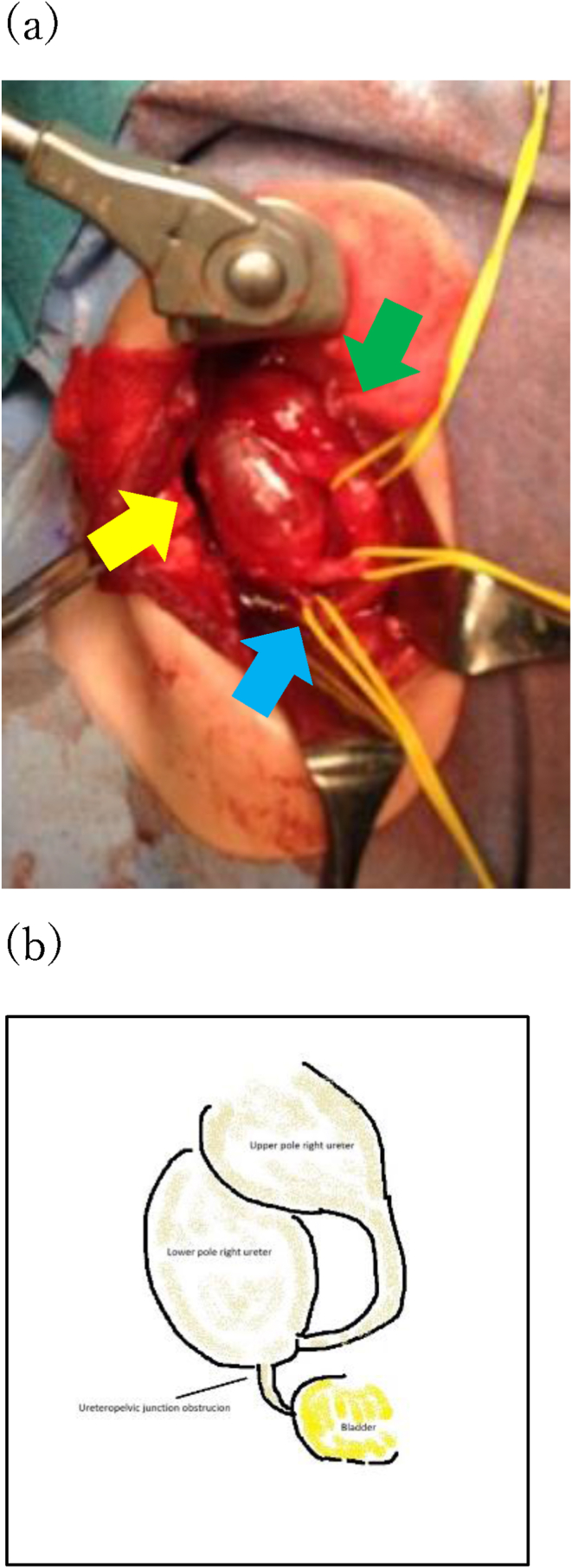
Intraoperative anatomical findings. The upper pole ureter (green arrow) drains into the dilated renal pelvis of the lower pole. The lower pole of the ureter exhibits uretero-pelvic junction obstruction, and the segment of the ureter between the lower pole and bladder is markedly narrow (blue arrow). Yellow arrow: lower pole right ureter. Green arrow: upper pole right ureter.

The ureteral stent was removed 1 month after surgery. The nephrostomy was clamped and then removed. The clamping time was gradually increased each day, and the nephrostomy catheter was finally removed 2 months after surgery. Subsequently, there were no urinary tract infections requiring hospitalization, and the dilation of the renal pelvis gradually improved. The dilation of the renal pelvis observed on abdominal ultrasonography gradually improved (Fig. 4). As of 5 years after surgery, there have been no hospitalization events due to urinary tract infections, and no complications, such as stenosis or renal atrophy, have been observed.

**Fig. 4:**
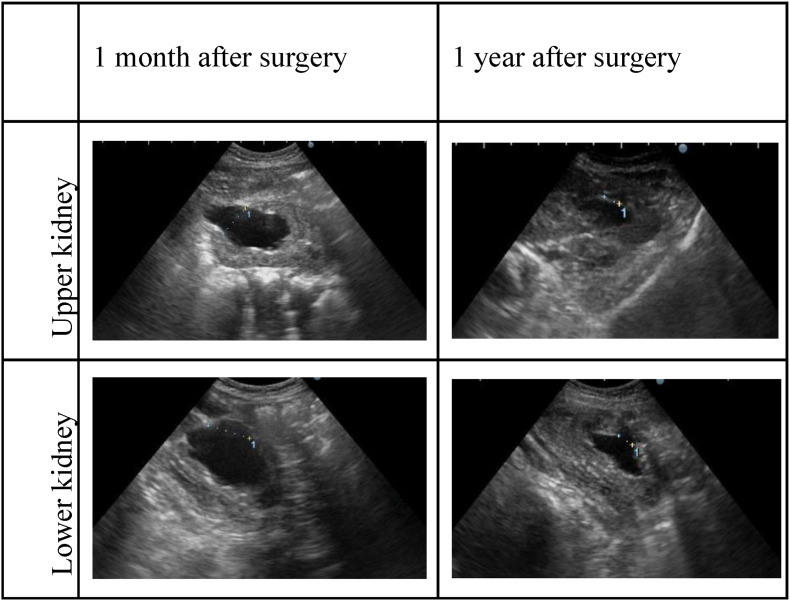
Changes in postoperative hydronephrosis findings. Gradual improvement in renal pelvic dilatation was observed in both the upper and lower poles over the course of 1 year postoperatively.

## 3 Discussion

We performed pyeloplasty for UPJO associated with a horseshoe kidney. The success rate of laparoscopic pyeloplasty for general UPJO is reported to be 94-98%^4^; however, there are few reports on the treatment outcomes for cases associated with horseshoe kidneys. In the present case, the duplicated collecting system had multiple strictures, and preoperative imaging did not provide a clear understanding of the anatomy of the urinary tract. Therefore, we opted for an open surgery. Although other surgical techniques could be considered, our anastomosis method resulted in no significant postoperative complications and a gradual improvement in hydronephrosis over time.

Cases of horseshoe kidneys with duplicated collecting systems have been previously reported; however, they are rare. Mirzazdeh et al. reported that, at the time of their report, there were only five cases of horseshoe kidneys with completely duplicated collecting systems, and four of them had ureteroceles in the upper pole ureter.^5^ Although there have been several reports of incomplete duplication cases from the time of publication of their report to the present, the exact incidence of cases with horseshoe kidneys combined with duplicated collecting systems is unknown. There has also been a case report of treatment of cases of incompletely duplicated collecting systems with UPJO^6^; however, such anatomical occurrences are considered even rarer.

Renal pyelic fusion has been described in association with horseshoe kidneys; however, its exact incidence remains unknown. There have been reports of cases in which the renal pelvises were fused and converged into a single ureter.^7^, ^8^ In contrast, Bhat et al. reported a case in which the renal pelvis of right kidney in a normal position was fused with the left renal pelvis, with their ureter not converged.^9^ To the best of our knowledge, this is the first reported case of a horseshoe kidney with renal pyelic fusion and the presence of ureters on both sides. The fusion of the renal pelvis was accidently discovered through ureterography, and this is considered extremely rare.

We encountered a rare anatomical case of a horseshoe kidney with a duplicated collecting system and fused renal pelvis. Although its incidence is low, it is necessary to consider this variation as a possible association with horseshoe kidneys. Appropriate surgical intervention is required depending on the patient's condition.

## CRediT authorship contribution statement

**Yoshinori Ikehata:** Writing – original draft. **Naotaka Nishiyama:** Writing – review & editing. **Hiroshi Kitamura:** Writing – review & editing.

## Informed consent

Written informed consent was obtained from the patient.

## Conflict of interest

The authors declare no conflict of interest.

## References

[1] Weizer A.Z., Silverstein A.D., Auge B.K. (2003). Determining the incidence of horseshoe kidney from radiographic data at a single institution. J Urol.

[2] Cascio S., Sweeney B., Granata C., Piaggio G., Jasonni V., Puri P. (2002). Vesicoureteral reflux and ureteropelvic junction obstruction in children with horseshoe kidney: treatment and outcome. J Urol.

[3] Partin A.W., Dmochowski R.R., Kavoussi L.R., Peters C. (2021).

[4] Bove P., Ong A.M., Rha K.H., Pinto P., Jarrett T.W., Kavoussi L.R. (2004). Laparoscopic management of ureteropelvic junction obstruction in patients with upper urinary tract anomalies. J Urol.

[5] Mirzazadeh M., Richards K.A. (2011). Complete duplication of collecting system in a horseshoe kidney presenting with recurrent urinary tract infections: report of an exceedingly rare congenital anomaly and review of literature. Sci World J.

[6] Tsuru N., Mugiya S., Kurita Y., Sato S., Hirano Y. (2016). Laparoscopic pyeloplasty for ureteropelvic junction obstruction in an incompletely duplicated collecting system in a patient with a horseshoe kidney. Urol Case Rep.

[7] Yesilli C., Erdem O., Akduman B., Erdem Z., Gundogdu S., Mungan N.A. (2003). Horseshoe kidney with pyelic fusion and crossed single ureter. J Urol.

[8] Enganti B., Chitekela N., Nallabothula A.K. (2013). Renal pyelic fusion with crossed solitary ureter: case report and review of literature. Int J Urol.

[9] Bhat S., Paul F., Doddamani S.C. (2015). Renal pyelic fusion anomaly. Ind J Urol.

